# The Combined Utilization of Epithelial Thickness and Tomographic Parameters in Keratoconus Detection

**DOI:** 10.1155/joph/6647993

**Published:** 2025-03-18

**Authors:** Abdelrahman Salman, Mayank Nanavaty, Rana Omran, Marwan Ghabra, Obeda Kailani, Rafea Shaaban, Taym Darwish, Buraa Kubaisi, Zein Baradi, Mohamed Khallouf, Omar Badla

**Affiliations:** ^1^Department of Ophthalmology, Latakia University, Latakia, Syria; ^2^Syrian Scientific Board of Ophthalmology, Health Ministry, Damascus, Syria; ^3^Department of Ophthalmology, University Hospitals Sussex NHS Foundation Trust, London, UK; ^4^Department of Cornea, Eye Surgical Hospital, Health Ministry, Damascus, Syria; ^5^Department of Ophthalmology, Whipps Cross University Hospital, Leytonstone, London, UK; ^6^Department of Ophthalmology, King's College Hospital NHS Foundation Trust, London, UK; ^7^Department of Ophthalmology, Tartous University, Tartous, Syria; ^8^Department of Research and Development, Tartous University, Tartous, Syria; ^9^Department of Research, Bascom Palmer Eye Institute, University of Miami, Miami, Florida, USA

## Abstract

**Purpose:** To evaluate the performance of the anterior segment optical coherence tomography (AS-OCT) and Scheimpflug–Placido tomographic (SPT) indices in differentiating suspect keratoconus (SKC) from normal (N).

**Methods:** In this cross-sectional study, one eye of each patient was scanned on AS-OCT and SPT. Following, 5 regression analysis models were developed: Model 1: combined AS-OCT corneal thickness (CT); Model 2: combined AS-OCT epithelial thickness (ET); Model 3: Models 1 + 2; Model 4: SPT indices (symmetry index front [SIf], symmetry index back [SIb], keratoconus vertex back [KVb], and minimum CT [ThkMin]); Model 5: Models 3 + 4. The areas under the curve (AUC) of receiver operator characteristic (ROC) curves were compared across groups to estimate their performance accuracy.

**Results:** Two hundred and five N eyes, 56 SKC eyes, and 89 keratoconus (KC) eyes were scanned with AS-OCT and SPT. For Models 1 and 2, no individual metric yielded an AUC > 0.84. For Model 3, the AUC was 0.96 (R2 Nagelkerke: 0.69) with 90.28% cases correctly identified as SKC. For Model 4, the AUC was 0.99 (R2 Nagelkerke: 0.87), with 95.58% of cases correctly classified as SKC. For Model 5, the diagnostic accuracy did not improve compared to Model 4.

**Conclusion:** Both combined AS-OCT indices and combined SPT indices provided excellent diagnostic power to differentiate SKC from N eyes. The combined SPT model showed a superior value compared to the AS-OCT model. Furthermore, combined AS-OCT and SPT data did not perform better than the combined SPT data alone.

## 1. Introduction

Keratoconus (KC) is a progressive corneal disease that results in corneal thinning, protrusion, irregular astigmatism and visual impairment [1]. The prevalence of KC ranges between 0.2 and 4.790 per 100,000 persons [2, 3], with a recent prevalence of 1.43% reported from a university student population in Syria [4]. Screening for KC in refractive surgery candidates is particularly important due to the risk of postoperative ectasia in subjects with undiagnosed early KC [5]. Therefore, detecting KC at its earlier stage is the primary goal in preventing postlaser-assisted in situ keratomileusis (LASIK) ectasia. Although the diagnoses of clinical KC do not raise challenges, many screening tests are needed to detect the early stages of this disease. The term suspect KC (SKC) should be reserved for corneas that exhibit subtle topographic signs of KC (such as inferior (I) steepening or asymmetrical bowtie) without evidence of clinical KC in either eye [6]. However, positive topographic findings are not necessarily indicative of KC. Some corneal anomalies induced by corneal scarring, dry eye, or hard contact lenses may yield false positives on videokeratoscopy. Similarly, the negative classification may not indicate the absence of an early form of KC [7]. The corneal epithelium can thin over the location of corneal steepening, thus masking early stromal irregularities [8]. Several studies have suggested that adding epithelial thickness (ET) data may allow for better sensitivity and specificity for the detection of KC compared to topographical evaluation alone [9–11].

Our previous study evaluated the diagnostic accuracy of topographic and tomographic indices obtained with Sirius corneal tomography (CSO, Italy) for detecting KC and SKC [7]. Our results demonstrated higher accuracy of posterior corneal surface indices than anterior surface–derived indices for detecting SKC, with the highest power reported for symmetry index back (SIb). We further investigated the application of corneal thickness (CT) and ET indices obtained by anterior segment optical coherence tomography (AS-OCT) in detecting KC and SKC, where our results revealed that most evaluated indices were excellent in distinguishing between KC and normal (N) corneas. In contrast, no indice was sufficient to distinguish between SKC and N corneas [12]. This study aimed to evaluate the diagnostic ability of combined Cirrus AS-OCT–based indices and combined Sirius Scheimpflug–Placido–based topography (SPT) indices, individually and in combination, in distinguishing between SKC eyes and N eyes.

## 2. Materials and Methods

This was a cross-sectional observational study. The study followed the tenets of the Declaration of Helsinki, and Institutional Review Board's approval was obtained (approval number: TUH-06023). Participants were recruited from the outpatient clinics at the Eye Surgical Hospital (the largest ophthalmology hospital in Syria), Ministry of Health, Damascus, Syria. All participants were at least 18 years old and provided written informed consent.

The diagnosis of SKC and KC was confirmed by two experienced cornea specialists (RO and BK). The diagnosis of KC was made if there was (a) an irregular cornea determined by distorted keratometry mires and/or distortion of the retinoscopic reflex or one of the following slit-lamp findings: Vogt's striae, Fleischer's ring, Munson's sign, iron ring, Rizzuti's sign, and apparent focal corneal bulging and thinning, or corneal scarring consistent with KC, in addition to (b) a positive Sirius software indicator [7]. The diagnosis of the SKC group was made by the absence of clinical (keratometric, retinoscopic, or biomicroscopic) signs of KC in either eye and corrected distance visual acuity (CDVA) of 20/20 or better, in addition to positive Sirius software indicator [7]. The repeatability of Sirius tomography in detecting KC and SKC is demonstrated in previous studies [7, 13]. N participants were recruited from patients seeking refractive surgery consultation. All N eyes had N Sirius software classification, with no signs of KC on slit-lamp and CDVA equal to or better than 20/20. One eye was randomly included if both eyes were KC-positive or SKC–positive. Only data from one eye were randomly included from N subjects. Patients were recruited consecutively, and all subjects meeting the inclusion criteria were included in the analysis.

Exclusion criteria were corneal hydrops, previous corneal or ocular surgery (such as cataract, glaucoma, corneal collagen cross-linking, excimer laser surgery, intrastromal corneal rings, and phakic intraocular lens), a history of ocular or systemic disease (e.g., uveitis, glaucoma, atopic dermatitis, and connective tissue disorders), lactation, or pregnancy. Contact lens wearers in the preceding three months were also excluded.

Preoperative assessment of all patients included autorefractokeratometry (SEIKO CO, GR-3500KA, Japan), uncorrected distance visual acuity (UDVA), CDVA, slit-lamp biomicroscopy, Goldmann applanation tonometry, and fundoscopy. The Sirius (CSO, Florence, Italy) Scheimpflug–Placido–based imaging carried out corneal topography and tomography (SPT). Simulated keratometry (k flat, k steep, and k average), symmetry index front (SIf), KC vertex front (KVf), SIb, KC vertex back (KVb), minimum corneal thickness (CT) (ThKmin), and asphericity (*Q*) values at 6, 7, 8, 9, and 10 mm zones were recorded.

In line with our previous study [12], CT and ET for the central (0–2 mm) and paracentral (2–5 mm) were measured using the Cirrus 5000 AS-OCT (Carl Zeiss Meditec, Germany) technology. The cornea-specific lens was used for the pachymetry map and HD cornea scans. Cirrus scans were captured with the subjects sitting, with their gaze fixed towards the fixation light target. Two scans were obtained for each eye by a single operator with 60 s rest periods to optimize the tear film rest, and average values were registered. Cirrus HD-OCT software (Version 10.0) provides average automated CT from three concentric ring-shaped zones: 0–2 mm central, 2–5 mm paracentral, and 5–7 mm midperipheral, and ET from four zones: 0–2 mm central, 2–5 mm paracentral, 5–7 mm midperipheral, and 7–9 mm peripheral. CT and ET are also presented for specific octants of the cornea: superior (S), I, temporal (T), nasal (N), SN, ST, IT, and IN within the concentric zones. Based on our previous study [12], CT and ET parameters that best characterize KC are evaluated and presented in Table 1.

### 2.1. Statistical Analysis

Statistical analysis was performed using MedCalc Statistical Software, Version 19.5.3 (MedCalc Software bv, Ostend, Belgium) and SPSS software (Version 17, SPSS Inc, Chicago, Illinois, United States of America). The normality of continuous data was checked using the Kolmogorov–Smirnov test. As most data were not normally distributed, a comparison was performed with the two groups using the Mann–Whitney *U* test. Receiver operating characteristic (ROC) curves were used to distinguish KC and SKC from N corneas. These curves were obtained by plotting sensitivity against specificity, calculated for each value observed. The area under the curve (AUC) measures discrimination, which is the test's ability to accurately classify eyes with and without disease. An area of 1.0 represents a perfect test, whereas an area of 0.5 represents a poor test. A stepwise logistic regression was performed to define the key parameters involved in detecting SKC. The Hosmer–Lemeshow adjustment was used to assess the overall goodness of fit of the model, and R2 Cox and Snell and R2 Nagelkerke were used to study the variance rate explained by the model's indices.

The performance of five logistic regression analysis (LRA) models to differentiate SKC eyes from N eyes was evaluated as follows:

  Model 1: used combined AS-OCT CT indices
  Model 2: used combined AS-OCT ET indices
  Model 3: was an addition of Model 1 and Model 2
  Model 4: used SPT indices
  Model 5: added Models 3 and 4

*p* values less than 0.05 were considered statistically significant.

## 3. Results

The demographic data for each group is presented in Table 2. Three hundred and fifty eyes were included and separated into three groups: N (*n*: 205), SKC (*n*: 56), and KC (*n*: 89). The mean age was only significantly different between the N and KC groups (*p* < 0.01). The mean sphere and cylinder values were significantly higher in the KC group compared to the N and SKC groups (*p* < 0.01), and the mean cylinder value was significantly higher in the SKC group compared to the N group (*p* < 0.03).

### 3.1. Cirrus AS-OCT CT

The CT data significantly differed between the KC and the N groups for all evaluated parameters (*p* < 0.01). All CT data are presented in Table 1.  Table 3 presents the statistically different parameters between the SKC and N groups.

### 3.2. Cirrus AS-OCT ET

The ET data (Table 1) were statistically different between the KC group and the N group for all ET parameters (*p* < 0.01), except Max ET (0_2) (*p*=0.89). ET parameters with statistical differences between the SKC and the N groups are presented in Table 3.

### 3.3. Sirius SPT

All evaluated topographic and tomographic parameters were statistically different between the K and N groups (*p* < 0.01). SPT indices with statistical differences between the SKC and the N groups are presented in Table 4.

### 3.4. Multivariate Models and LRA

AS-OCT and SPT indices were selected for stepwise multivariate models, and their AUC, sensitivity, and specificity in discrimination of SKC from N corneas and K from N ones are shown in Table 5.

For AS-OCT, four CT indices were defined as key parameters to detect SKC: Min CT (2_5), Avg CT (2_5), Max CT (2_5), and Min–Max CT (2_5), with the highest AUC to detect SKC and KC seen for Min CT (2_5), 0.84 and 0.975, respectively. The LRA defined four ET indices as key parameters to detect SKC: Min ET (0_2), Min ET (2_5), Min–Max ET (2_5), and SN–IT ET (2–5). While ET (0_2) and Min–Max ET (2_5) were excellent in differentiating KC from N corneas (AUC > 0.9, both), none of the ET indices were strong enough to distinguish SKC from N cornea (AUC < 0.8), with the highest AUC seen for Min ET (0_2) and Min–Max ET (2_5) (AUC = 0.74, both). For SPT, LRA defined 4 indices as key parameters to detect SKC: SIf, SIb, KVb, and ThKmin. While the highest strength to detect KC was registered for KVb (AUC = 0.988), the highest strength to differentiate SKC from N cornea was seen for SIb (AUC = 0.951).

The performance of the LRA models for differentiating SKC eyes from N eyes is presented in Table 6.

  Model 1: The LRA of combined AS-OCT CT indices reached an AUC of 0.95 (R2 Cox and Snell = 0.44 and R2 Nagelkerke = 0.67), with 88.71% of cases correctly classified as SKC.
  Model 2: The ability of Model 2 (combined AS-OCT ET data) was lower than that of Model 1, with an AUC of 0.82 (R2 Cox and Snell = 0.20 and R2 Nagelkerke = 0.31). In contrast, the percentage of cases correctly identified by Model 2 was 81.12%.
  Model 3: The output values of the AS-OCT logistic regression based on CT and ET data reached an AUC of 0.96 (R2 Cox and Snell = 0.45 and R2 Nagelkerke = 0.69), a sensitivity of 74.55%, a specificity of 97%, and an ability of 90.28% to classify cases as SKC correctly.
  Model 4: The highest diagnostic power to differentiate SKC from N eyes was seen for the combined SPT, with an AUC of 0.99 (Cox and Snell = 0.57 and R2 Nagelkerke = 0.87) and 95.58% cases correctly classified as SKC eyes.
  Model 5: There were no differences between the performances of Model 5 and Model 4 alone.

## 4. Discussion

Several studies have tried to introduce a specific criterion to detect early forms of KC [14–16]. This has gained particular importance for ruling out suspect cases when screening candidates for refractive surgery to minimize the risk of post-LASIK corneal ectasia. This study aimed to present new predictive models for the detection of SKC. We investigated the diagnostic performance of combined AS-OCT CT data, combined AS-OCT ET data, combined SPT data, and combined AS-OCT and SPT data. Our findings demonstrated that combined AS-OCT and SPT data allowed the generation of multivariate models that accurately differentiated between SKC corneas and N corneas.

Corneal stromal and ET changes associated with KC are well-documented features of the disease [8, 12, 17, 18]. In our previous study, we used a high-definition AS-OCT to compare the diagnostic ability of CT and ET parameters in KC and SKC cases [12]. While all evaluated CT and ET parameters were strong enough to differentiate KC eyes from N eyes (AUC > 0.9), none of these parameters were sufficient enough to differentiate SKC from N eyes (AUC < 0 .8 for all indices), with the highest diagnostic power registered for CT (AUC = 0.76) and Min–Max ET (2_5) (AUC = 0.71). Elkitkat et al. [19] investigated the diagnostic accuracy of keratometric, pachymetric, elevation, KC summary, aberrations, and ET indices obtained with MS-39 AS-OCT (CSO, Italy). Their results demonstrated that ET parameters were not among the highest AUC in differentiating between KC and N eyes. These findings are consistent with ours; the diagnostic power of CT indices was higher than ET indices, with the highest diagnostic power to detect SKC reported for Min CT (2_5) and Min–Max ET (2_5) and AUC = 0.84 and 0.744, respectively. These findings are consistent with those reported by Heideri et al. [20], where they found that the diagnostic accuracy was significantly higher for stromal thickness in SKC (AUC: 0.75) than other individual corneal layers.

Our results demonstrated that combined AS-OCT ET (Model 2) reached only 0.82 AUC, while combined AS-OCT CT (model 1) achieved an accuracy of 0.95 AUC. However, by combining CT and ET data (Model 3), AUC improved to 0.96. Four individual AS-OCT–based indices were defined to be included in Model 3: Avg CT (2–5), Max CT (2–5), Min–Max CT (2–5), and Min ET (2–5). These parameters were registered at the 2–5 mm paracentral zones. However, this is not unexpected since the early KC cone is more pronounced in the paracentral zone. In line with a previous report [20], these parameters showed significantly lower values in the SKC group than in the N group. Yücekul, Dick, and Taneri [18] used a two-step decision tree to combine CT and ET parameters obtained with AS-OCT (Zeiss Cirrus 5000 HD) to differentiate subclinical KC from N corneas, where they achieved 90.4% sensitivity. Yang et al. [17] developed a two-step decision tree method that combined CT and ET parameters derived from AS-OCT (Avanti, Optovue, Inc. Fermont, California, United States of America). They reached a sensitivity of 73.7% in differentiating forme fruste KC from N corneas. In this study, combined AS-OCT CT and ET indices (Model 3) showed a sensitivity of 81.12% in distinguishing SKC eyes from N eyes.

Corneal tomography is currently the best tool to diagnose early KC, which evaluates the anterior and posterior cornea and produces CT data. This study's combined SPT logistic regression model Mmodel 4) showed the highest diagnostic power to distinguish SKC from N corneas. This model utilized both anterior corneal data (SIf), and posterior corneal data (SIb and KVb) and pachymetric data (ThkMin).

Gharieb et al. [21] used Sirius tomography to find the best indices for differentiating between thin, N corneas, FFKC, and early KC. The anterior apex curvature had the highest ability to detect early KC. Shetty et al. [22] evaluated several indices derived from SPT to differentiate SKC from N corneas, where they reported the highest sensitivity for SIf and the highest specificity for SIb. Among different parameters, Heidari et al. [23] found that SPT SIb was the best diagnostic parameter with an AUC of 0.908 for detecting subclinical KC. Our previous study identified SIb as the best diagnostic parameter for detecting SKC, with an AUC of 0.86 [7]. Golan et al. [24] used Pentacam HR (Oculus, Germany) to examine different parameters obtained from the posterior corneal surface. They identified the posterior elevation index as the most sensitive parameter to detect early KC [24]. Recent investigations have identified the posterior corneal surface as a site at which early changes occur during the progression of KC [25, 26].

Arbelaez et al. [13] utilized a support vector machine (SVM) to define a new classifier for diagnosing KC based on corneal measurements provided by SPT. The classifier was trained to consider the indices obtained from the anterior and posterior corneal surfaces or only from the anterior corneal surface. Using the data from both anterior and posterior corneal surfaces and pachymetry allowed the SVM to increase its sensitivity from 75.2% to 92.0% in eyes with subclinical KC. The Belin–Ambrosio enhanced ectasia display (BAD) on Pentacam (Oculus, Germany) combines both anterior and posterior elevation data and pachymetric data to screen for ectatic changes [27]. Golan et al. [28] used LRA to determine the best model combination of corneal metrics measured by a dual Scheimpflug/Placido device (Galilei G4, Ziemer Ophthalmic Systems AG, Port, Switzerland). Among the nine metrics included, pachymetry values were the most impactful metrics. The model yielded an AUC of 0.96 to distinguish unaffected eyes (similar to the forme fruste KC eyes) in patients with asymmetric KC from N eyes [28]. The most significant tomography-derived indices across models included anterior and posterior corneal asymmetry, posterior elevation, and pachymetric metrics similar to those used in our model.

Adding AS-OCT–based ET and stromal thickness data to tomographic data may improve early KC detection. Hwang et al. [29] used LRA to evaluate Scheimpflug (Pentacam) and AS-OCT (RTVue, Optovue, Inc., Fremont, United States of America) measurements for unaffected eyes of asymmetric KC patients' detection. They reported that combined AS-OCT CT and ET metrics performed (AUC: 0.96) better than combined Scheimpflug metrics (AUC: 0.86). A combination of indices from AS-OCT and Scheimpflug technologies performed optimally (AUC: 1) in their study population. However, the results may differ from the method used to analyze the data. Shi et al. [10] used the Pentacam HR system and ultra-high-resolution optical coherence tomography (UHR-OCT) imaging data to distinguish between subclinical KC and N eyes. Using the Pentacam HR system alone or UHR-OCT alone, the logistic regression classifier showed an AUC of 0.74 (Pentacam HR system) and an AUC of 0.90 (UHR-OCT); the neural network classifier reached an AUC = 0.68 (Pentacam HR system) and an AUC = 0.88 (UHR-OCT). After combining the Pentacam HR data and UHR-OCT data, the neural network classifier reached an AUC of 0.93, while the classifier did not improve (AUC = 0.9) for the logistic regression classifier. This study's LRA demonstrated that combined SPT metrics were S to combined AS-OCT–based metrics in differentiating between SKC and N eyes, AUC O.99 versus AUC 0.96, respectively. However, by combining SPT and AS-OCT–based metrics, the diagnostic accuracy was not improved compared to SPT alone. Consistent with our findings, the diagnostic accuracy was not improved compared to a single machine–derived logistic regression model, as the precision accuracy of combined SPT data was almost similar to the diagnostic accuracy of combined SPT and AS-OCT data.

Our study had several limitations. The absence of biomechanical indices contributed to KC is considered a limitation of our study. Moreover, the current Cirrus 5000 OCT software does not provide the epithelial pattern standard deviation parameter, which has previously demonstrated a high diagnostic power in detecting early KC. The patient's age is an important variable in the progression and stage of the disease. The significant difference in the age averages between the N group and the KC group is considered as another limitation of our study. Future studies with similar age averages between groups would be useful to examine whether a statistically different result emerges with subgroup analyses. Our study is a cross-sectional study but to our knowledge this study gives insightful information which is not yet available in the literature.

In conclusion, our study demonstrated that combined SPT–based Sirius and AS-OCT–based Cirrus indices provided excellent power to differentiate SKC from N eyes. However, The SPT features showed S value compared to the AS-OCT features. Furthermore, multiple instrument–combined indices did not perform better than single instrument–derived indices.

## Figures and Tables

**Table 1:** List of Cirrus variables used in the study.

	Description
*CT variables*	
Min CT (0_2)	The minimum corneal thickness at the 0–2 mm central zone
Avg CT (0_2)	The average corneal thickness at the 0–2 mm central zone
Max CT (0_2)	The maximum corneal thickness at the 0–2 mm central zone
Min–Max CT (0_2)	The minimum corneal thickness minus the maximum corneal thickness at the 0–2 mm corneal zone
Min CT (2_5)	The minimum corneal thickness at the 2–5 mm paracentral zone
Avg ET (2_5)	The average epithelial corneal thickness at the 2–5 paracentral zone
Max CT (2_5)	The maximum corneal thickness at the 2–5 mm paracentral zone
Min–Max CT (2_5)	The minimum corneal thickness minus the maximum corneal thickness at the 2–5 mm paracentral zone
S–I CT (2_5)	The mean corneal thickness of the superior octant minus that of the inferior octant at the 2–5 mm paracentral zone
SN–IT CT (2_5)	The difference between mean superonasal and mean inferotemporal corneal thickness at the 2–5 mm paracentral zone
CCT	The central corneal thickness

*ET variables*	
Min ET (0_2)	The minimum epithelial thickness at the 0–2 mm central zone
Avg ET (0_2)	The average epithelial thickness at the 0–2 mm central zone
Max ET (0_2)	The maximum epithelial thickness at the 0–2 mm central zone
Min–Max ET (0_2)	The minimum epithelial thickness minus the maximum corneal thickness at the 0–2 mm corneal thickness
Min ET (2_5)	The minimum epithelial thickness at the 2–5 mm paracentral zone
Avg ET (2_5)	The average epithelial corneal thickness at the 2–5 paracentral zone
Max ET (2_5)	The maximum epithelial corneal thickness at the 2–5 mm paracentral zone
Min–Max ET (2_5)	The minimum epithelial corneal thickness minus the maximum thickness at the 2–5 mm paracentral zone
S–I ET (2_5)	The average epithelial thickness of the superior octant minus that of the inferior octant at the 2–5 mm paracentral zone
SN–IT ET (2_5)	The difference between mean superonasal and mean inferotemporal corneal epithelial thickness at the 2–5 mm paracentral zone
CET	The thickness of the epithelium at the central point

**Table 2:** Demographic characteristics of groups.

	N	SKC	KC	*p* value		
Patients, (*n*)	205	56	89	Normal vs. SKC	Normal vs. KC	SKC vs. KC
Age, (mean ± SD)	27.25 ± 5.43	25.5 ± 7.28	22.8 ± 7.78	0.359	**< 0.0001**	0.062
Sphere, D (mean ± SD), (range)	−0.41 ± 1.83, (−7.75; 3.5)	−0.11 ± 1.37, (−5.5; 2.75)	−2.05 ± 2.8, (−12; 3)	1	**< 0.0001**	**< 0.0001**
Cylinder, D (mean ± SD), (range)	−0.73 ± 1.14, (−5.5; 3.75)	−1.7 ± 1.82, (−7.75; 1.5)	−3.87 ± 3.02, (−12; 1.5)	**0.003**	**< 0.0001**	**< 0.0001**

*Note:* Mann–Whitney test was used to compare the difference between means of each two samples. Values in bold reached statistical significance (*p* < 0.05).

Abbreviations: D = diopter; KC = keratoconus; *n* = number; N = normal; SD = standard deviation; SKC = suspect keratoconus.

**Table 3:** Cirrus parameters with statistically significant differences between the N group and the SKC group (mean ± SD in microns).

Patients, (*n*)	N				SKC				KC			
	205				56				89			
Pachymetry (0_2)	Mean	SD	Range		Mean	SD	Range		Mean	SD	Range	
Min CT (0_2)	535	34	456	645	489	31	415	546	440	48	257	538
Avg CT (0_2)	541	34	461	655	500	32	425	560	463	44	307	559
Max CT (0_2)	554	34	480	671	516	34	437	585	493	41	344	592
Min–Max CT (0_2)	−18	7	−35	0	−27	11	−59	−9	−51	29	−227	0
Min CT (2_5)	536	34	456	644	489	31	415	546	431	72	70	527
Avg CT (2_5)	560	34	483	671	522	33	444	586	497	38	352	586
Max CT (2_5)	601	37	515	718	565	38	474	655	556	41	486	764
Min–Max CT (2_5)	−62	20	−103	0	−76	20	−133	−42	−119	81	−520	0
SN–IT CT (2_5)	29	13	−16	63	36	15	−2	80	50	27	−34	135
CCT	539	34	462	652	497	32	422	558	457	46	287	554

*Epithelial thickness*												
Min ET (0_2)	49	4	39	60	45	4	37	58	38	5	30	52
Avg ET (0_2)	51	4	42	67	48	5	40	63	44	5	36	57
Min–Max ET (0–2)	−3	2	−20	0	−5	3	−11	−1	−12	8	−50	0
Min ET (2_5)	45	3	37	53	43	3	37	55	37	5	21	51
Min–Max ET (2_5)	−7	4	−26	0	−11	4	−23	−4	−19	10	−58	0
SN–IT ET (2_5)	0	2	−8	6	2	4	−10	10	8	6	−5	23
CET	51	4	43	67	48	5	38	63	43	7	4	62

Abbreviations: Avg CT (0_2) = average corneal thickness at 0–2 mm central zone; Avg CT (2_5) = average corneal thickness at 2–5 mm paracentral zone; Avg ET (0_2) = average epithelial thickness at 0–2 mm central zone; CCT = central corneal thickness; CET = central epithelial thickness; KC = keratoconus; Max CT (0_2) = maximum corneal thickness at 0–2 mm central zone; Max CT (2_5) = maximum corneal thickness at 2–5 mm paracentral zone; Min CT (0_2) = minimum corneal thickness at 0–2 mm central zone; Min CT (2_5) = minimum corneal thickness at 0–2 mm central zone; Min ET (0_2) = minimum epithelial thickness at 0–2 mm central zone; Min ET (2_5) = minimum corneal thickness at 2–5 mm paracentral zone; Min–Max CT (0_2) = minimum–maximum corneal thickness at 0–2 mm central zone; Min–Max CT (2_5) = minimum–maximum corneal thickness at 2–5 mm paracentral zone; Min–Max ET (0–2) = minimum–maximum epithelial thickness at 0–2 mm central zone; Min–Max ET (2_5) = minimum–maximum epithelial thickness at 2–5 mm paracentral zone; *n* = number; N = normal; SKC = suspect keratoconus; SN–IT CT (2_5) = superonasal–inferotemporal corneal thickness at 2–5 paracentral zone; SN–IT ET (2_5) = superonasal–inferotemporal epithelial thickness at 2–5 paracentral zone.

**Table 4:** Sirius parameters with statistically significant differences between the N group and the SKC group.

(*n*)	N				SKC				KC			
	205				56				89			
	Mean	SD	Range		Mean	SD	Range		Mean	SD	Range	
SIf (D)	0.02	0.43	−1.2	1.13	1.30	0.93	−0.44	3.26	6.20	4.84	−0.6	35
KVf (μm)	3.81	1.58	−0.04	15	8.85	4.25	3	19	32.12	20.47	0.7	151
SIb (D)	0.08	0.32	−0.25	3.3	0.51	0.29	0.09	1.16	1.94	3.04	−0.6	28
Kvb (μm)	10.15	2.89	4	25	21.54	9.08	7	42	70.69	41.07	1.4	294
ThkMin (μm)	534.84	34.34	452	652	485.52	35.23	417	564	436.41	54.12	255	529
Q-6 mm	−0.18	0.17	−1.38	0.4	−0.50	0.33	−1.09	−0.02	−1.18	0.69	−3	0.5
Q-7 mm	−0.22	0.17	−1.39	0.41	−0.51	0.31	−1.04	−0.03	−1.12	0.59	−2.6	0.3
Q-8 mm	−0.30	0.16	−1.22	0.05	−0.54	0.29	−1.11	−0.09	−1.04	0.48	−2.1	0.1
Q-9 mm	−0.57	0.25	−1.47	0.4	−0.71	0.25	−1.17	−0.29	−1.09	0.51	−3	0.3

*Note:* μm = micron; Q = asphericity.

Abbreviations: D = diopter; KC = keratoconus; KVb = keratoconus vertex back; KVf = keratoconus vertex front; mm = millimeter; *n* = number; N = normal; SD = standard deviation; SIb = symmetry index back; SIf = symmetry index front; SKC = suspect keratoconus; ThkMin = minimum corneal thickness.

**Table 5:** Variables selected into multivariate models by logistic regression analysis and their AUC, sensitivity, and specificity in discrimination of suspect keratoconus from normal corneas and keratoconus from normal ones.

	Cutoff value		AUC		Sensitivity (%)		Specificity (%)	
	Normal vs. SKC	Normal vs. KC	Normal vs. SKC	Normal vs. KC	Normal vs. SKC	Normal vs. KC	Normal vs. SKC	Normal vs. KC
*Corneal thickness (μm)*								
Min CT (2_5)	≤ 521	≤ 493	0.84	0.975	85.71	95.29	62.69	89.12
Avg CT (2_5)	≤ 539	≤ 539	0.784	0.902	71.43	92.94	71.5	71.5
Max CT (2_5)	≤ 576	≤ 575	0.756	0.825	67.86	78.82	73.58	73.58
Min–Max CT (2_5)	≤ −77	≤ −87	0.672	0.886	48.21	74.16	81.95	93.66

*Epithelial thickness (μm)*								
Min ET (0_2)	≤ 46	≤ 43	0.742	0.948	64.29	88.24	72.82	93.33
Min ET (2_5)	≤ 42	≤ 41	0.714	0.927	53.57	88.24	80	85.64
Min–Max ET (2_5)	≤ −10	≤ −11	0.744	0.905	58.93	85.39	84.88	88.78
SN–IT ET (2–5)	> 0	> 4	0.678	0.914	64.29	75.29	61.03	96.92

*Sirius parameters*								
SIf	> 0.57	> 13	0.901	0.965	78.57	94.25	91.22	100
SIb	> 0.2	> 0.4	0.951	0.951	83.93	93.1	91.67	99.02
KVb	> 13	> 18	0.893	0.988	80.36	98.85	89.22	99.51
ThkMin	≤ 487	≤ 488	0.838	0.961	58.93	88.51	94.12	93.14

*Note:* μm = micron.

Abbreviations: AUC = areas under the curve; Avg CT (2_5) = average corneal thickness at 2–5 mm paracentral zone; KC = keratoconus; KVb = keratoconus vertex back; Max CT (2_5) = maximum corneal thickness at 2–5 mm paracentral zone; Min CT (2_5) = minimum corneal thickness at 2–5 mm paracentral zone; Min ET (0_2) = minimum epithelial thickness at 0–2 mm central zone; Min ET (2_5) = minimum epithelial thickness at 2–5 mm paracentral zone; Min–Max CT (2_5); minimum–maximum corneal thickness at the paracentral zone; Min–Max ET (2_5) = minimum–maximum epithelial thickness at 2–5 mm paracentral zone; SIb = symmetry index back; SIf = symmetry index front; SKC = suspect keratoconus; SN–IT ET (2–5) = superonasal–inferotemporal epithelial thickness at 2–5 mm paracentral zone; ThkMin = minimum corneal thickness; vs = versus.

**Table 6:** Performance of the logistic regression analysis models for differentiating suspect keratoconus eyes from normal eyes.

**Model 1 (combined CT AS-OCT data)**											
	**Odds ratio**	**95% CI**		***p* value**	**Cox and Snell** **R** ^2^	**Nagelkerke** **R** ^2^	**AUC**	**95% CI**	**Percent of cases correctly classified**	**Sensitivity (%)**	**Specificity (%)**

Min CT (2_5)	0.9978	0.9978	0.9978	< **0.0001**	0.44	0.67	0.95	0.906 to 0.968	88.71%	64	96
Avg CT (2_5)	1.78	1.45	2.18	< **0.0001**							
Max (CT (2_5)	0.54	0.54	0.54	< **0.0001**							
Min–Max CT (2_5)	0.65	0.65	0.65	< **0.0001**							

**Model 2 (combined ET AS-OCT data)**											
	**Odds ratio**	**95% CI**		***p* value**	**Cox and Snell** **R** ^2^	**Nagelkerke** **R** ^2^	**AUC**	**95% CI**	**Percent of cases correctly classified**	**Sensitivity (%)**	**Specificity (%)**

Min ET (0_2)	0.82	0.75	0.90	< **0.0001**	0.20	0.31	0.82	0.768 to 0.867	81.12%	30.36	95
Min–Max ET (2–5)	0.83	0.76	0.91	**0.0001**							
SN–IT ET (2–5)	1.19	1.05	1.36	**0.0067**							

**Model 3 (combined CT and ET AS-OCT data)**											
	**Odds ratio**	**95% CI**		**p** **value**	**Cox and Snell** **R** ^2^	**Nagelkerke** **R** ^2^	**AUC**	**95% CI**	**Percent of cases correctly classified**	**Sensitivity (%)**	**Specificity (%)**

Avg CT (2–5)	1.75	1.42	2.16	< **0.0001**	0.45	0.69	0.96	0.914 to 0.973	90.28%	74.55	97
Max CT (2–5)	0.54	0.44	0.68	< **0.0001**							
Min–Max CT (2–5)	0.65	0.56	0.75	< **0.0001**							
Min ET (2–5)	0.82	0.70	0.95	**0.008**							

**Model 4 (combined Scheimpflug**–**Placido data)**											
	**Odds ratio**	**95% CI**		**p** **value**	**Cox and Snell** **R** ^2^	**Nagelkerke** **R** ^2^	**AUC**	**95% CI**	**Percent of cases correctly classified**	**Sensitivity (%)**	**Specificity (%)**

SIf	93.54	9.03	969.31	**0.0001**	0.57	0.87	0.99	0.968 to 0.998	95.58%	87.27	98
SIb	11.17	2.81	44.41	**0.0006**							
KVb	1.51	1.25	1.84	< **0.0001**							
ThkMin	0.93	0.90	0.96	< **0.0001**							

**Model 5 (combined AS-OCT and Scheimpflug**–**Placido data)**											
	**Odds ratio**	**95% CI**		**p** **value**	**Cox and Snell** **R** ^2^	**Nagelkerke** **R** ^2^	**AUC**	**95% CI**	**Percent of cases correctly classified**	**Sensitivity (%)**	**Specificity (%)**

SIf	89.87	8.82	915.32	**0.0001**	0.57	0.87	1.00	0.966 to 0.998	95.97%	87.5	98
SIb	10.85	2.73	43.11	**0.0007**							
KVb	1.51	1.24	1.83	< **0.0001**							
ThKmin	0.93	0.90	0.96	< **0.0001**							

*Note:* Values in bold reached statistical significance (*p* < 0.05).

Abbreviations: AS-OCT = anterior segment optical coherence tomography; AUC = areas under the curve; Avg CT (2_5) = average corneal thickness at 2–5 mm paracentral zone; CI = confidence interval; CT = corneal thickness; ET = epithelial thickness; KVb = keratoconus vertex back; Max CT (2_5) = maximum corneal thickness at 2–5 mm paracentral zone; Min CT (2_5) = minimum corneal thickness at 2–5 mm paracentral zone; Min ET (2–5) = minimum epithelial thickness at 2–5 mm paracentral zone; Min–Max CT (2–5) = minimum–maximum corneal thickness at 2–5 mm paracentral zone; SIb = symmetry index back; SIf = symmetry index front; ThkMin = minimum corneal thickness.

## Data Availability

The datasets generated and analyzed during the current study are available from the corresponding author (Abdelrahman Salman) on reasonable request.

## References

[1] Salman A., Ghabra M., Darwish T. R., Kailani O., Ibrahim H., Ghabra H. (2022). Corneal Higher-Order Aberration Changes After Accelerated Cross-Linking for Keratoconus. *BMC Ophthalmology*.

[2] Al Bdour M., Sabbagh H. M., Jammal H. M. (2024). Multi-Modal Imaging for the Detection of Early Keratoconus: A Narrative Review. *Eye and Vision*.

[3] Hashemi H., Heydarian S., Hooshmand E. (2020). The Prevalence and Risk Factors for Keratoconus: A Systematic Review and Meta-Analysis. *Cornea*.

[4] Salman A., Darwish T., Ghabra M. (2022). Prevalence of Keratoconus in a Population-Based Study in Syria. *Journal of Ophthalmology*.

[5] Randleman J. B. (2010). Evaluating Risk Factors for Ectasia: What Is the Goal of Assessing Risk?. *Journal of Refractive Surgery*.

[6] Salman A., Kailani O., Marshall J. (2022). Evaluation of Anterior and Posterior Corneal Higher Order Aberrations for the Detection of Keratoconus and Suspect Keratoconus. *Tomography*.

[7] Salman A., Darwish T., Ali A., Ghabra M., Rafea S. (2021). Sensitivity and Specificity of Sirius Indices in Diagnosis of Keratoconus and Suspect Keratoconus. *European Journal of Ophthalmology*.

[8] Reinstein D. Z., Gobbe M., Archer T. J., Silverman R. H., Coleman D. J. (2010). Epithelial, Stromal, and Total Corneal Thickness in Keratoconus: Three-Dimensional Display With Artemis Very-High Frequency Digital Ultrasound. *Journal of Refractive Surgery*.

[9] Alio Del Barrio J. L., Eldanasoury A. M., Arbelaez J., Faini S., Versaci F. (2024). Artificial Neural Network for Automated Keratoconus Detection Using a Combined Placido Disc and Anterior Segment Optical Coherence Tomography Topographer. *Translational Vision Science and Technology*.

[10] Shi C., Wang M., Zhu T. (2020). Machine Learning Helps Improve Diagnostic Ability of Subclinical Keratoconus Using Scheimpflug and OCT Imaging Modalities. *Eye and Vision*.

[11] Silverman R. H., Urs R., RoyChoudhury A., Archer T. J., Gobbe M., Reinstein D. Z. (2017). Combined Tomography and Epithelial Thickness Mapping for Diagnosis of Keratoconus. *European Journal of Ophthalmology*.

[12] Salman A., Mazzotta C., Kailani O. (2023). Diagnostic Accuracy of Corneal and Epithelial Thickness Map Parameters to Detect Keratoconus and Suspect Keratoconus. *Journal of Ophthalmology*.

[13] Arbelaez M. C., Versaci F., Vestri G., Barboni P., Savini G. (2012). Use of a Support Vector Machine for Keratoconus and Subclinical Keratoconus Detection by Topographic and Tomographic Data. *Ophthalmology*.

[14] Castro-Luna G., Perez-Rueda A. (2020). A Predictive Model for Early Diagnosis of Keratoconus. *BMC Ophthalmology*.

[15] Niazi S., Jiménez-García M., Findl O. (2023). Keratoconus Diagnosis: From Fundamentals to Artificial Intelligence: A Systematic Narrative Review. *Diagnostics*.

[16] Yekta A., Hashemi H., Ostadimoghaddam H. (2023). Anterior and Posterior Corneal Higher-Order Aberrations in Early Diagnosis and Grading of Keratoconus. *Clinical and Experimental Optometry*.

[17] Yang Y., Pavlatos E., Chamberlain W., Huang D., Li Y. (2021). Keratoconus Detection Using OCT Corneal and Epithelial Thickness Map Parameters and Patterns. *Journal of Cataract and Refractive Surgery*.

[18] Yucekul B., Dick H. B., Taneri S. (2022). Systematic Detection of Keratoconus in OCT: Corneal and Epithelial Thickness Maps. *Journal of Cataract and Refractive Surgery*.

[19] Elkitkat R. S., Rifay Y., Gharieb H. M., Ziada H. E. A. (2022). Accuracy of the Indices of MS-39 Anterior Segment Optical Coherence Tomography in the Diagnosis of Keratoconic Corneas. *European Journal of Ophthalmology*.

[20] Heidari Z., Mohammadpour M., Hajizadeh F., Fotouhi A., Hashemi H. (2024). Corneal Layer Thickness in Keratoconus Using Optical Coherence Tomography. *Clinical and Experimental Optometry*.

[21] Gharieb H. M., Abdelatif M. K., Gharieb H. M., Othman I. S. (2024). Early, Forme Fruste Keratoconus and Normal Thin Cornea, Evaluation of Sensitive Parameters by Combined Placido Scheimpflug Topography. *European Journal of Ophthalmology*.

[22] Shetty R., Rao H., Khamar P. (2017). Keratoconus Screening Indices and Their Diagnostic Ability to Distinguish Normal From Ectatic Corneas. *American Journal of Ophthalmology*.

[23] Heidari Z., Mohammadpour M., Amanzadeh K., Fotouhi A., Fotouhi A. (2021). Evaluation of Corneal Topographic, Tomographic and Biomechanical Indices for Detecting Clinical and Subclinical Keratoconus: A Comprehensive Three-Device Study. *International Journal of Ophthalmology*.

[24] Golan O., Hwang E. S., Lang P. (2018). Differences in Posterior Corneal Features Between Normal Corneas and Subclinical Keratoconus. *Journal of Refractive Surgery*.

[25] Cunha A. M., Correia P. J., Alves H. (2021). Keratoconus Enlargement as a Predictor of Keratoconus Progression. *Scientific Reports*.

[26] Ribeiro M., Barbosa C., Correia P. (2022). Best Fit Sphere Back and Adjusted Maximum Elevation of Corneal Back Surface as Novel Predictors of Keratoconus Progression. *Clinical Ophthalmology*.

[27] Bamdad S., Sedaghat M. R., Yasemi M., Vahedi A. (2020). Sensitivity and Specificity of Belin Ambrosio Enhanced Ectasia Display in Early Diagnosis of Keratoconus. *Journal of Ophthalmology*.

[28] Golan O., Piccinini A. L., Hwang E. S. (2019). Distinguishing Highly Asymmetric Keratoconus Eyes Using Dual Scheimpflug/Placido Analysis. *American Journal of Ophthalmology*.

[29] Hwang E. S., Perez-Straziota C. E., Kim S. W., Santhiago M. R., Randleman J. B. (2018). Distinguishing Highly Asymmetric Keratoconus Eyes Using Combined Scheimpflug and Spectral-Domain OCT Analysis. *Ophthalmology*.

